# Ceramide lipid-based nanosuspension for enhanced delivery of docetaxel with synergistic antitumor efficiency

**DOI:** 10.1080/10717544.2016.1225853

**Published:** 2017-05-13

**Authors:** Tianqi Wang, Lixia Feng, Shaomei Yang, Yongjun Liu, Na Zhang

**Affiliations:** School of Pharmaceutical Science, Shandong University, Ji’nan, People’s Republic of China

**Keywords:** Ceramide, docetaxel, lipid-based nanosuspension, co-delivery, combination therapy

## Abstract

Ceramide (CE), a bioactive lipid with tumor suppression, has been widely used as a drug carrier and enhancer for cancer therapy. CE-based combination therapy was prone to be attractive in cancer therapy. In our previous study, the combination of CE and docetaxel (DTX) was proved to be an effective strategy for cancer therapy. To further improve the antitumor efficiency of DTX, the CE lipid-based nanosuspensions (LNS) was prepared for the delivery of DTX to exhibit synergistic therapeutic effect. The enhanced delivery and synergistic therapeutic effect of DTX-loaded CE-LNS (CE + DTX-LNS) were evaluated. CE + DTX-LNS exhibited spherical or ellipsoidal shape, uniform particle size distribution (108.1 ± 3.8 nm), sustained release characteristics and good stability *in vitro*. Notably, CE + DTX-LNS could effectively co-localize CE and DTX into same tumor cell and subsequently play synergistic cell damage effect compared with CE-LNS + DTX-LNS (*p* < 0.05). The *in vivo* fluorescence imaging results showed that CE + DTX-LNS could effectively prolong the *in vivo* circulation time and enhance the accumulation in tumor sites. Moreover, the antitumor efficacy of CE + DTX-LNS observed in B16 murine melanoma model was 93.94 ± 2.77%, significantly higher than that of CE-LNS, DTX-LNS, Duopafei® (*p* < 0.01) and CE-LNS + DTX-LNS (*p* < 0.05), respectively, demonstrating that co-delivery of CE and DTX into same tumor cell was the basis for enhanced synergistic therapeutic effect. Furthermore, histological examination of Blank-LNS showed no visible tissue toxicity compared to normal saline. Consequently, CE-LNS could effectively delivery DTX and CE + DTX-LNS exhibit synergistic inhibition of tumor growth due to the co-localization of CE and DTX. CE-LNS hold great potential to be an appropriate carrier for CE-based combination chemotherapy.

## Introduction

Combination therapy was widely used in current clinical cancer treatment (Peters et al., 2000; Shukla et al., 2014). For example, paclitaxel/doxorubicin and paclitaxel/carboplatin have been widely studied and used on treatment of lung cancer, showing good therapeutic effects (Zhang et al., 2015; Wang et al., 2016). Different from traditional combination of cytotoxic drugs, combining bioactive agent with cytotoxic drug could produce additive or even synergistic activity without generating excessive toxicity, providing a promising direction for combination therapy (Tkaczuk, 2009).

Among various bioactive agents, ceramide (CE) has been widely recognized and has become a hot research topic (Liu et al., 2010; Cho et al., 2011; Ma et al., 2016). CE is a naturally occurring membrane sphingolipid, which was considered as a lipid second messenger that mediate diverse cellular effects, such as cell growth, differentiation and death (Czubowicz & Strosznajder, 2014; Chi le et al., 2015). Different from cytotoxic drugs, CE could specially kill cancer cells and did not produce additional toxicity to normal cells or immune system (Selzner et al., 2001; Li & Zhang, 2015). Besides, CE could enhance the response of cytotoxic drugs, demonstrating great potential in combination therapy (Ji et al., 2010; Zhu et al., 2011). In our previous study, it was proved that CE combined with docetaxel (DTX) (CE + DTX) at molar ratio of 1:2 could produce the greatest synergistic antitumor effect in murine melanoma B16 cell and human breast carcinoma MCF-7 cell (Feng et al., 2014). Specially, CE could target the microfilament actin, leading to the polymerization and destruction of actin cytoskeleton, while DTX could target and disrupt the microtubules cytoskeleton. Notably, CE + DTX could cause a synergistic destruction of cytoskeleton, resulting in significantly higher apoptosis and significantly higher arrest in G2/M phase comparing with either agent alone (*p* < 0.01). Thus, it is valuable to carry out further studies to develop novel combination therapeutic product based on CE + DTX.

Combination therapeutic agents should be simultaneously delivered to the same tumoral cell to exert their synergistic effect (Kemp et al., 2016). It was proven to be challenging to develop delivery systems which can efficiently encapsulate the two therapeutic agents and successfully deliver them to the targeted sites via systemic administration. To improve the delivery efficiency and synergistic effect of combined agents, different kinds of nanovectors, including nanoparticles, polymeric micelles and liposomes has been introduced (Fleige et al., 2012; Tang et al., 2016). For example, the multi-functional micelles for synergistic co-delivery of doxorubicin (DOX) and paclitaxel (PTX) that Lin-Yue Lanry Yung and his coworkers prepared (Duong & Yung, 2013).

Considering PTX is a hydrophobic drug and CE is a natural sphingolipid, lipid-based nanocarrier was suitable to co-delivery PTX and CE (Ma et al., 2016). As a natural lipid, CE was regarded as drug carrier material to construct CE lipid-based nanosuspensions (LNS). CE-LNS were capable of loading PTX with synergistic treatment effect. LNS is colloidal dispersion with dispersed nanosized drug particles in the range of 100 nm to 500 nm in an aqueous media (Zhang & Zhang, 2013). Using phospholipids as stabilizer against self-aggregation, DTX-loaded CE-LNS (CE + DTX-LNS) holds the advantages of both lipid-based nanocarriers and nanosuspensions: (1) no drug leakage problems; (2) without excessive loading of foreign materials or organic solvent; (3) formulate compounds that are insoluble in both water and oil; (4) drug loading is high and the administration volume is significantly reduced (Wang et al., 2011; Zhang & Chen, 2012).

**Figure 1.**
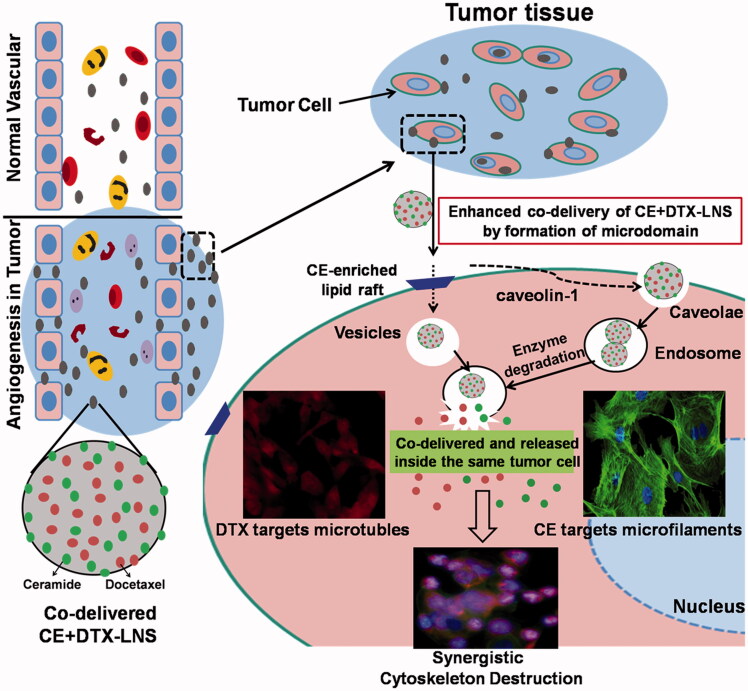
Scheme of the synergistic mechanisms of nanosized CE + DTX-LNS. After intravenous administration, CE + DTX-LNS was accumulated at tumor site through EPR effect and internalized into same cell through vesicles and caveolae mediated endocytosis. Subsequently, CE and DTX with different anti-cancer mechanisms were released independently from CE + DTX-LNS inside the same cancer cell and killed the cancer cell in an enhanced synergistic manner.

In CE + DTX-LNS system, CE served not only as the loaded drug but also the lipid material. Therefore, it was relatively easy to control the optimal combination ratio of CE and DTX by adjusting the dose of CE, paving the way for precise combination design *in vivo* cancer therapy (Aryal et al., 2011; Kratz & Warnecke, 2012). Moreover, CE partly existed on the surface of CE + DTX-LNS, was expected to promote the formation of microdomain or CE-enriched lipid raft on cell membrane, promoting signal transduction and protein transport, favoring to the cellular uptake of CE + DTX-LNS via the vesicles and caveolae-mediated endocytosis (Figure 1) (Li et al., 2010; Li & Zhang, 2015; Su et al., 2015).

In the present study, to promote synergistic combination cancer treatment effect, DTX-loaded CE-LNS was prepared using high pressure homogenization method. The *in vitro* synergistic antitumor effect was confirmed by MTT assay and Caspase-3 activity study. The co-delivery efficiency of CE and DTX into same tumor cell was examined by fluorescence microscope and flow cytometry. After that, the *in vivo* synergistic anti-tumor efficacy of CE + DTX-LNS was investigated in B16 tumor bearing mice, and the co-delivery efficiency along with the *in vivo* synergistic therapeutic effect were simultaneously explored. Furthermore, the biodistribution of CE + DTX-LNS were studied by fluorescence imaging technology in B16 tumor bearing mice, and the tissue toxicity of Blank-LNS (LNS without both CE and DTX loaded) after administration was studied by histological examination.

## Materials and methods

### Materials

Injectable soya lecithin (phosphatidylcholine accounts for 95%, pH 5.0–7.0) was provided by Shanghai Taiwan Pharmaceutical Co., Ltd. (Shanghai, China). Glycerol was purchased from Sinopharm Co., Ltd. (Shanghai, China). Ceramide (CE) and NBD-ceramide (NBD-CE) were obtained from Avanti Polar Lipids, Inc. (Alabaster, AL). Docetaxel (DTX) was provided by Chenxin Pharmaceutical Co., Ltd. (Jining, China). Rhodamine-DTX (Rho-DTX) was synthesized by Na Zhang’s team. 3-(4,5-Dimethylthiazol-2-yl)-2,5-diphenyltetrazolium bromide (MTT) was purchased from Solarbio (Shanghai, China). Caspase-3 Activity Assay Kit was obtained from Beyotime (Shanghai, China). Hoechst 33342 was purchased from Invitrogen by Life Technologies (Carlsbad, CA). All the other chemicals and reagents used were of analytical purity grade or higher, obtained commercially.

### Cell lines and cell culture

Murine melanoma cell line B16 was purchased from Chinese Academy of Sciences (Shanghai, China). Human breast carcinoma cell line MCF-7 was kindly provided by Institute of Immunopharmacology and Immunotherapy of Shandong University (Jinan, China). B16 and MCF-7 cell lines were cultured in RPMI-1640 media at 37 °C under 5% CO_2_. All the media were supplemented with 10% (v/v) fetal bovine serum from Sijiqing Co. Ltd, streptomycin at 100 μg/mL and penicillin at 100 U/mL.

### Preparation of lipid-based nanosuspension (LNS)

In order to effectively deliver CE and DTX, the LNS loaded with both CE and DTX (CE + DTX-LNS) was prepared by high pressure homogenization. Briefly, soya lecithin (1%, w/v) and glycerol (2%, w/v) were dissolved in water to obtain the aqueous surfactant solution. To keep the optimal combination molar ratio of CE and DTX (CE: DTX = 1:2), DTX powder (0.1%, w/v) and CE powder (0.025%, w/v) were added proportionally to the aqueous surfactant solution (Feng et al., 2014). And then the mixture was totally mixed under high speed shearing to obtain the drug coarse suspension. The coarse suspension was then circulated through the high pressure homogenizer (NS1001L, Niro Soavi S.P.A., Italy) until an equilibrium size was reached. After sterile filtration, the freshly prepared CE + DTX-LNS was dispensed into glass vials, 5% mannitol (w/v) was added subsequently to the vials as a lyoprotectant and frozen for 24 h at −80 °C. These vials were then transferred to a freeze dryer (LGJ0.5; Beijing Four-Ring Scientific Instrument Co, Beijing, China) and dried for 48 h at −40 °C and a pressure of 0.5 mbar to get the lyophilized CE + DTX-LNS.

LNS loaded with CE (CE-LNS) (0.025%, w/v), LNS loaded with DTX (DTX-LNS) (0.1%, w/v) and LNS without both CE and DTX loaded (Blank-LNS) were prepared as controls, respectively, with the same method introduced above.

### Characterization of CE + DTX-LNS

The morphology of CE + DTX-LNS was examined by transmission electron microscopy (TEM) (JEM-120 0EX, Japan). Samples were prepared by placing a drop of CE + DTX-LNS onto a copper grid and air dried, following negative staining with a drop of 3% sodium phosphotungstate for contrast enhancement (Sun et al., 2008). The mean particle size and zeta potential of CE + DTX-LNS were analyzed by Delsa^TM^ Nano Submicron Particle Size and Zeta Potential Particle Analyzer photon correlation spectroscopy (PCS) (Beckman Coulter, Brea, CA). All the measurements were carried out in triplicates. The average particle size was expressed in volume mean diameter.

### Stability of CE + DTX-LNS

To determine the stability of CE + DTX-LNS under physiologically relevant conditions, the lyophilized CE + DTX-LNS was dispersed in different media: PBS, complete cell culture media (10% fetal bovine serum, v/v) and PBS containing human plasma (10%, v/v), respectively. The particle size of CE + DTX-LNS was measured by PCS after 4, 8, 12, 24 and 48 h of incubation, respectively. The stability of CE + DTX-LNS was demonstrated by the absence of macroscopic aggregates and unchanged particle size. Moreover, the long-term physical stability of the lyophilized CE + DTX-LNS was evaluated at −20 °C ± 2 °C, 4 °C ± 2 °C and 25 °C ± 2 °C. The changes in morphology and particle size were recorded over a period of 3 months.

### *In vitro* release studies

The *in vitro* release profile of DTX and CE from CE + DTX-LNS was conducted by a dialysis bag diffusion method, respectively. CE was replaced by NBD-CE (10%, w/w) to study the *in vitro* release profile of CE. Typically, lyophilized NBD-CE + DTX-LNS was suspended in 2 mL of de-ionized water (final concentration of DTX and NBD-CE was 100 μg/mL and 25 μg/mL, respectively) and placed into a pre-swelled dialysis bag (MWCO = 8–12 kDa). The bag was incubated in 15 mL release media (PBS containing 0.5% of Tween-80) at 37 ± 0.5 °C under horizontal shaking (Yanasarn et al., 2009). At predetermined time points (0.5, 1, 2, 4, 8, 12, 24, 36 and 48 h), 2 mL of release media was withdrawn and replaced with an equal volume of fresh media. The concentration of DTX in dialyzate was analyzed by HPLC method while the concentration of NBD-CE was analyzed fluorometrically at λ_Ex _= 460 nm and λ_Em _= 530 nm. Release profiles of Duopafei®, DTX-LNS, NBD-CE-Solution (NBD-CE dissolved in DMSO and diluted with PBS), NBD-CE-LNS were simultaneously reported for comparison.

### Cellular uptake of CE + DTX-LNS

To investigate the co-delivery of DTX and CE into tumor cells via the co-delivered CE + DTX-LNS, CE and DTX were replaced by NBD-CE and Rho-DTX separately to prepare NBD-CE + Rho-DTX-LNS as described in preparation of LNS. B16 cells were seeded in a 24-well culture plate and incubated for overnight at 37 °C, 5% CO_2_, followed by adding the NBD-CE + Rho-DTX-LNS (final concentration of NBD-CE and Rho-DTX was 1 μg/ml and 4 μg/ml, respectively). After 4 h incubation, culture media was removed and cells were washed twice with the pre-colded PBS. The cells were directly observed under a fluorescence microscope (BX40, Olympus, Tokyo, Japan).

For quantitative study, MCF-7 and B16 cells after incubation with NBD-CE + Rho-DTX-LNS were washed twice with PBS at predetermined time intervals (0.5, 2 and 4 h, respectively), trypsinized with trypsin-EDTA solution, and analyzed on a FACS Calibur flow cytometer (BD Biosciences, Franklin Lakes, NJ). The results were analyzed using WinMDI 2.9 software. Cells treated with the physical mixture of NBD-CE-LNS plus Rho-DTX-LNS (NBD-CE-LNS + Rho-DTX-LNS) with the same amount of drug were served as control.

### Anti-proliferation test *in vitro*

The anti-proliferation effects of CE-LNS (No DTX-LNS), DTX-LNS (No CE-LNS), CE-LNS + DTX-LNS (with DTX-LNS 20 μM or with CE-LNS 10 μM) at different molar ratio and CE + DTX-LNS were investigated by MTT assay on B16 and MCF-7 cells, respectively. Briefly, cells in the exponential growth phase were counted and added into the 96-multiwell plate. Cells were allowed to adhere overnight at 37 °C and 5% CO_2_ before drug treatments. CE-LNS stock solution (0.25 mg/ml) and DTX-LNS stock solution (1 mg/ml) were serially diluted with pH 7.4, 0.01 M phosphate-buffered saline (PBS) at a concentration of 0.5–40 μM, respectively, and added proportionally to the 96-well plate simultaneously. After 48 h incubation, the samples were measured by a microplate reader (FL600, Bio-Tek Inc., Winooski, VT). The relative cell viability (%) compared to control cells was calculated by [Abs_sample_/Abs_control_] × 100%.

### Apoptosis effect of CE + DTX-LNS *in vitro*

To quantitatively investigate the apoptosis effect induced by CE + DTX-LNS, the Caspase-3 activity on B16 and MCF-7 cells was evaluated by Caspase-3 Activity Assay Kit. Briefly, Caspase-3 was activated by treating cells with CE + DTX-LNS (10 μM CE plus 20 μM DTX), while cells incubated with CE-LNS (10 μM), DTX-LNS (20 μM) and CE + DTX-solution (CE + DTX dissolved in DMSO and diluted with PBS, 10 μM CE plus 20 μM DTX) were set as controls. After 24 h or 48 h incubation, the cells with different treatments were harvested and resuspended with 1 × lysis buffer in the assay kit for 15 min at 0 °C, and then centrifuged at 20 000 *g* for 15 min to collect the supernatant containing cell extracts. To initiate the enzymatic reaction, the fresh cell extracts with different treatments were transferred into the well of a 96-well plate, respectively, followed by the addition of assay buffer and Caspase-3 substrate (Ac-DEVD-pNA). The absorbance was measured at 405 nm by a microplate reader. In order to quantify the Caspase-3 activity, the protein content of each drug treatment was evaluated by Bradford protein assay, following the manufacturer’s assay instructions.

### *In vivo* and *ex vivo* fluorescence imaging study

The biodistribution of CE + DTX-LNS was assessed in B16 tumor-bearing mice with *in vivo* fluorescence imaging technology. B16 tumor-bearing mice were intravenously injected with NBD-CE + Rho-DTX-LNS (at the dose of 0.16 mg/kg NBD-CE and 0.64 mg/kg Rho-DTX, respectively), while mice treated with NBD-CE + Rho-DTX-solution and normal saline (N.S) were served as control. NBD channel were taken with 460 nm excitation filter and 530 nm emission filter while Rhodamine channel taken with 550 nm excitation filter and 620 nm emission filter. At predetermined time points (1, 4 and 8 h, respectively), animals were placed in an IVIS light-tight chamber and anesthesia was maintained with 2.0% isoflurane. Image acquisition was performed at different time intervals on a Xenogen IVIS Lumina system (Caliper Life Sciences, Hopkinton, MA). Results were analyzed using Living Image 3.1 software (Caliper Life Sciences, Hopkinton, MA).

To quantitatively study the biodistribution of CE + DTX-LNS, mice were sacrificed at 4 h after administration for *ex vivo* study, and the heart, liver, spleen, lung, kidney and tumor were excised, washed with cold saline and observed using the Xenogen IVIS Lumina system. The total amounts of fluorescence (Epi) in different organs were quantitatively analyzed with Living Image 3.1 software at 30% threshold fluorescence intensity. Mice treated with NBD-CE + Rho-DTX-solution was served as controls. The excised organs from the control mice and the treated mice were placed in the same background, and then the fluorescent images of the organs were taken.

### *In vivo* antitumor efficacy of CE + DTX-LNS

The female Kunming mice (weight: 18–22 g, age: 6–8 weeks) were supplied by the Medical Animal Test Center of Shandong University (Jinan, China). All the experiments were carried out in compliance with the Animal Management Rules of the Ministry of Health of the People’s Republic of China (document number 55, 2001) and the Animal Experiment Ethics Review of Shandong University.

The *in vivo* antitumor efficacy evaluation was experimented in Kunming mice inoculated with B16 melanoma cells (3 × 10^5^) by subcutaneous injection at the right axillary space. Treatments were started after 10–12 days of the implantation. The mice with tumor volume of ∼100 mm^3^ were selected and this day was designated as ‘Day 0’. The mice were randomly assigned to 6 treatment groups: (1) CE + DTX-LNS (at 10 mg/kg DTX and 2.5 mg/kg CE, respectively, making sure the final combination molar ratio of CE and DTX was 0.5:1), (2) 2.5 mg/kg CE-LNS, (3) 10 mg/kg DTX-LNS, (4) 10 mg/kg Duopafei®, (5) N.S and (6) Blank-LNS (Liu et al., 2011). To make a further investigation of whether CE and DTX via the co-delivered CE + DTX-LNS would achieve synergistic anti-tumor effect, the dosage of CE-LNS, DTX-LNS and Duopafei® were subsequently doubled, respectively, and the physical mixture CE-LNS + DTX-LNS were served as control at the same time. As a result, 4 more treatment groups were generated: (7) 5 mg/kg CE-LNS, (8) 20 mg/kg DTX-LNS, (9) 20 mg/kg Duopafei® and (10) CE-LNS + DTX-LNS (at 10 mg/kg DTX and 2.5 mg/kg of CE, respectively) (Wang et al., 2012). All formulations were diluted with N.S and administered intravenously once every three days for 21 days (Wang et al., 2012).

All mice were labeled, and the tumors were measured every three days with calipers during the period of study. The tumor volume was calculated by the formula: *V* = (*W*^2 ^×^ ^*L*)/2, where *W* is the tumor measurement at the widest point and *L* is the tumor dimension at the longest point. Each animal was weighed at the time of administration, so that the dosage could be adjusted to achieve the required amounts reported. After 21 days, the animals were sacrificed and the tumor mass was harvested and weighed. The tumor inhibition ratio (TIR) could be defined as follows: TIR (%) = ([W_c_-W_t_]/W_c_) × 100%, where *W*_c_ and *W*_t_ stand for the average tumor weight for the control group and treatment group, respectively (Zhang et al., 2009).

### The histological observation

The safety of Blank-LNS was evaluated by the histological observation. Blank-LNS (the same dose as used in the *in vivo* antitumor efficacy evaluation studies) was injected into three female Kunming mice (18–22 g) through the tail vein every three days for three times, and mice treated with N.S were served as control. Ten days post injection, all animals were sacrificed. The liver, heart, spleen, lung and kidney were separated, washed twice with PBS and fixed in 4% formaldehyde for histological examination.

### Statistical analysis

All the studies were repeated a minimum of three times and measured at least in triplicate. Results were reported as means ± standard deviation (SD). Statistical significance was analyzed using the Student’s *t*-test. Differences between experimental groups were considered significantly when the *p*-value was less than 0.05 (*p* < 0.05).

## Results and discussion

### Characterization of CE + DTX-LNS

**Figure 2.**
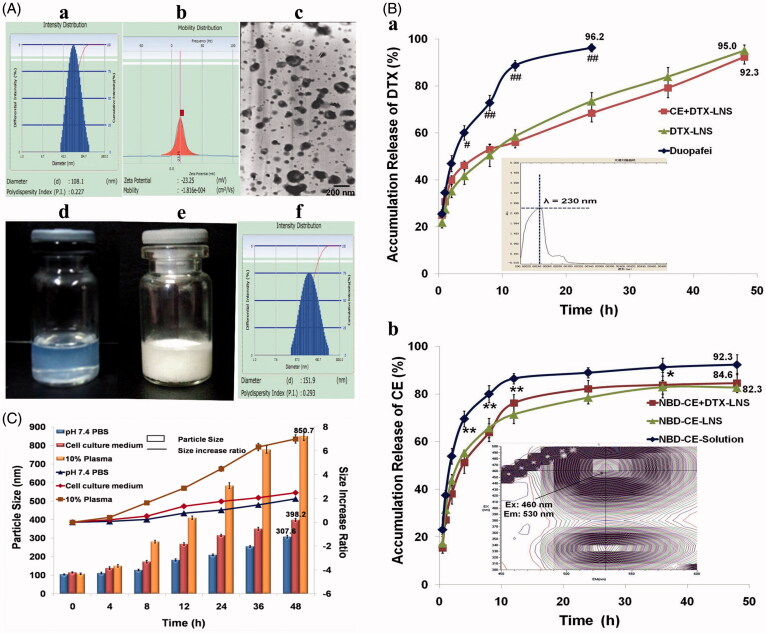
Characteristics of CE + DTX-LNS *in vitro*. (A) Particle size (a), zeta potential (b), TEM images (c) and photographs (d) of freshly prepared CE+DTX-LNS; Photographs (e) and particle size (f) of lyophilized CE + DTX-LNS. (B) In vitro drug release of CE+DTX-LNS (*n* = 3). (a) The release profile of DTX from CE + DTX-LNS, DTX-LNS and Duopafei® (b) The release profile of NBD-CE from NBD-CE + DTX-LNS, NBD-CE-LNS and NBD-CE-Solution. *p < 0.05, **p < 0.01, statistically significant difference between NBD-CE+DTX-LNS and NBD-CE-Solution; # *p* < 0.05, ##*p* < 0.01, statistically significant difference between CE+DTX-LNS and Duopafei®. (C) Stability of CE + DTX-LNS was evaluated by determination of particle size alteration overtime in different media: PBS, complete cell culture media and PBS containing 10% plasma (*n* = 3).

CE + DTX-LNS was prepared successfully with high pressure homogenization and the characteristics of CE + DTX-LNS were investigated and presented in Figure 2. The mean particle size of freshly prepared CE + DTX-LNS was 108.1 ± 3.8 nm (Figure 2(Aa) and the relative small size resulted in a state of transparent liquid (Figure 2(Ad) (Adkins et al., 2008). The polydispersity index of CE + DTX-LNS was 0.227 ± 0.086 (Figure 2(Aa), indicating a high homogenization of the particles. The zeta potential was -23.25 ± 4.17 mV (Figure 2(Ab), which was helpful to maintain the stability during transport in the blood circulation. The TEM micrograph of the fresh-prepared CE + DTX-LNS was shown in Figure 2(Ac). To keep long-term stability, the lyophilized formulation was prepared (Figure 2(Ae) and could be easily dissolved in physiological saline. The particle size of lyophilized CE + DTX-LNS was slightly increased to 151.9 ± 4.57 nm (Figure 2(Af) Moreover, there was no significant alteration on zeta potential and TEM micrograph, indicating that the freeze drying process has no impact on the characteristics of CE + DTX-LNS.

### Stability of CE + DTX-LNS

The stability of CE + DTX-LNS was studied by determination of the particle size alteration in different media, as shown in Figure 2(C). Within 4 h, the particle size of CE + DTX-LNS was slightly increased in PBS, complete cell culture media and PBS containing 10% plasma, indicating that CE + DTX-LNS could keep a good nanostructure during transport process. The good stability of CE + DTX-LNS could provide the opportunities for CE + DTX-LNS accumulating in tumor sites by EPR effect, which is beneficial for alleviation of side effects to bone, marrow, heart and kidney (Vicent & Duncan, 2006). Besides, after 48 h incubation, the particle size of CE + DTX-LNS was significantly increased in 10% plasma (*p* < 0.01) and cell culture media (*p* < 0.01), some macroscopic aggregates were even observed in 10% plasma media, which indicated strong destruction of nanostructure and complete release of drugs from CE + DTX-LNS.

### *In vitro* release of CE + DTX-LNS

For co-delivery of multiple anticancer agents, it is important to allow the nanocarrier to release the individual drugs independently, enabling them to attack cancer cells via their independent mechanisms of action and subsequently generate synergistic effect (Jager et al., 2013). Therefore, the release profile of DTX and NBD-CE from NBD-CE + DTX-LNS was investigated by the dynamic dialysis at 37 °C in pH 7.4 PBS to simulate conditions in blood circulation.

As shown in Figure 2(Ba), a significant lower release of DTX from CE + DTX-LNS (68.3 ± 3.57%, *p* < 0.01) and DTX-LNS (73.5 ± 3.68%, *p* < 0.01) were observed in comparison with Duopafei® (96.2 ± 1.68%) within 24 h. The sustained released profile of DTX from CE + DTX-LNS or DTX-LNS could successfully avoid an initial burst drug release and the acute toxicity. After 48 h, the remaining DTX loaded in CE + DTX-LNS and DTX-LNS was completely released up to 92.3 ± 2.98% and 95.0 ± 2.38% respectively, reaching the same level of Duopafei®. This phenomenon could be explained by the decrease in particle size, leading to an increased surface area of drug and possible better contact between nanosuspensions and dissolution media, which was finally favorable to the dissolution rate of DTX (Demetzos & Pippa, 2014). Similarly, NBD-CE was sustained and completely released from NBD-CE + DTX-LNS and NBD-CE-LNS. Especially, the release profile of DTX from CE + DTX-LNS displayed no difference with that from DTX-LNS, indicating that CE loaded in CE + DTX-LNS did not change the release profile of DTX. Similarly, DTX did not change the release profile of CE. Therefore, it was expected that the independence and sustained release of CE and DTX from CE + DTX-LNS would be favorable to the synergistic antitumor efficacy and the passive tumor-targeted drug delivery.

### Co-delivery of CE and DTX via CE + DTX-LNS

**Figure 3.**
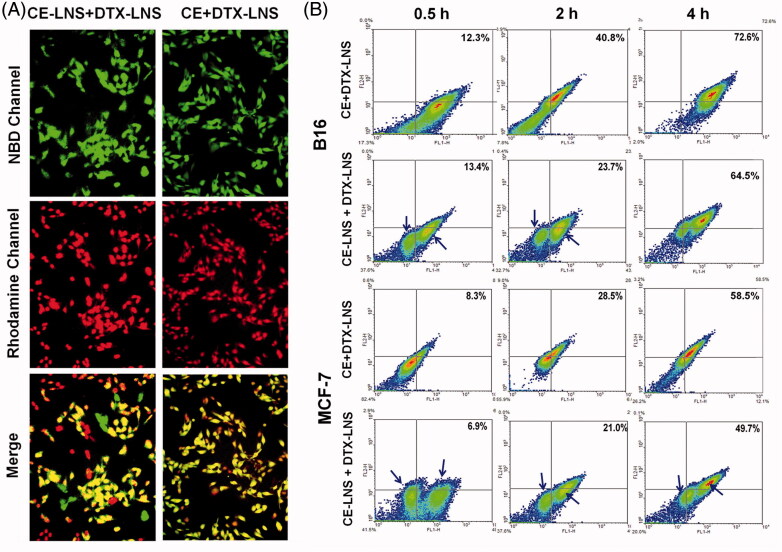
Co-delivery of CE and DTX via CE + DTX-LNS *in vitro*. (A) Co-delivery of CE and DTX via CE + DTX-LNS by fluorescence microscope (*n* = 3). B16 cells were incubated with NBD-CE and Rho-DTX labeled CE + DTX-LNS for 4 h while cells treated with NBD-CE-LNS + Rho-DTX-LNS were served as control (× 20). (B) Co-delivery of CE and DTX via CE + DTX-LNS by FACS analysis (*n* = 3). B16 and MCF-7 cells were incubated with NBD-CE and Rho-DTX labeled CE + DTX-LNS for 0.5, 2 and 4 h, respectively. Cells incubated with NBD-CE-LNS + Rho-DTX-LNS were served as control.

To investigate the co-localization of DTX and CE in tumor cells, the cellular uptake study of CE + DTX-LNS was carried out. As shown in Figure 3(A), fluorescence image exhibited strong and well co-localized Rho-DTX and NBD-CE fluorescence within tumor cell after incubated with NBD-CE + Rho-DTX-LNS, indicating the simultaneous uptake of both CE and DTX. In contrast, the physical mixture NBD-CE-LNS + Rho-DTX-LNS separately delivered NBD-CE and Rho-DTX to different cancer cells, leading to the less co-localized fluorescent signals, implying that CE-LNS + DTX-LNS was less effective for simultaneous delivery of CE and DTX *in vitro*.

To quantitatively evaluate the co-delivery efficiency of CE + DTX-LNS, the double-positive expressed MCF-7 and B16 cells incubated with NBD-CE + Rho-DTX-LNS were analyzed with FACS. Cells incubated with the physical mixture NBD-CE-LNS + Rho-DTX-LNS were served as control. As shown in Figure 3(B), both the cellular uptake of CE + DTX-LNS and CE-LNS + DTX-LNS were in dose-depended manner. The distribution of cancer cells treated with CE + DTX-LNS were relatively concentrated while the cells incubated with CE-LNS + DTX-LNS were obviously split into two populations. This phenomenon was probably caused by the separate delivery of CE and DTX to different cancer cells via CE-LNS and DTX-LNS, respectively, which further demonstrated the great advantage of CE + DTX-LNS in simultaneous delivery of CE and DTX into same tumor cell. Specially, majority of cells were located in the double-positive quadrant (72.6 ± 4.2% for B16 cells and 58.5 ± 3.7% for MCF-7 cells, respectively) after 4 h incubation with CE + DTX-LNS, which was significantly higher than that of CE-LNS + DTX-LNS (*p* < 0.05). This phenomenon was probably attributed to the interaction between CE and cell membrane. As a naturally occurring bioactive sphingolipid, CE partly existed on the surface of CE + DTX-LNS and was expected to promote the formation of microdomain or CE-enriched lipid raft on cell membrane, promoting signal transduction and protein transport, favoring to the cellular uptake of CE + DTX-LNS via the vesicles and caveolae-mediated endocytosis (Li et al., 2010; Li & Zhang, 2015; Su et al., 2015). In conclusion, CE + DTX-LNS could effectively deliver CE and DTX into same tumor cell and was favorable to the optimal dose schedule for CE-based combination therapy.

### Enhanced anti-proliferation effects of CE + DTX-LNS

**Figure 4.**
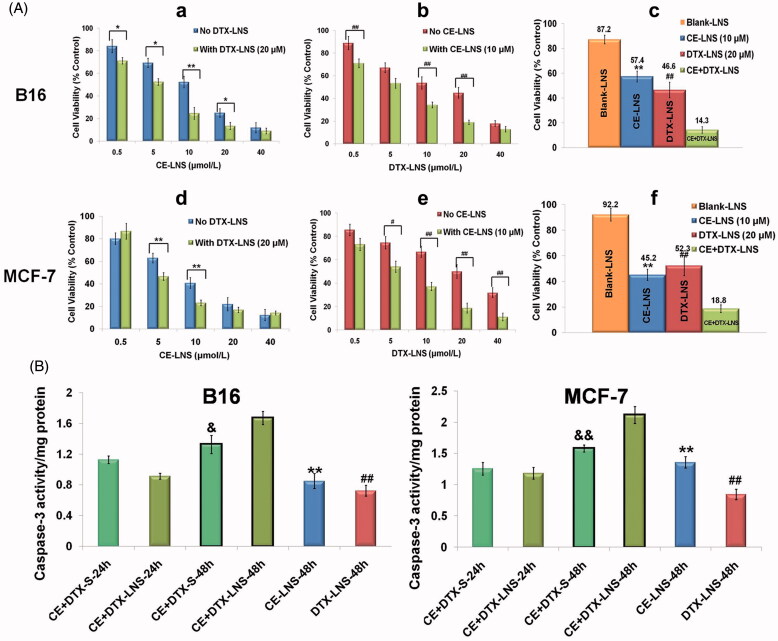
The anti-proliferation effect and apoptosis of CE + DTX-LNS *in vitro*.(A) Effects of different treatments on cell viabilities of B16 and MCF-7 cells, (*n* = 3). Cells were treated with CE-LNS (No DTX-LNS), DTX-LNS (No CE-LNS), CE-LNS + DTX-LNS (with DTX-LNS 20 μM or with CE-LNS 10 μM) at different molar ratio and CE + DTX-LNS. After 48 h incubation, cell viabilities were evaluated by MTT assay. * *p* < 0.05, ** *p* < 0.01, statistically significant difference between CE-LNS and CE + DTX-LNS; # *p* < 0.05, ## *p* < 0.01, statistically significant difference between DTX-LNS and CE + DTX-LNS. (B) Caspase-3 activity after different treatments on B16 and MCF-7 cells. ** *p* < 0.01, statistically significant difference between CE-LNS and CE + DTX-LNS; ## *p* < 0.01, statistically significant difference between DTX-LNS and CE + DTX-LNS; & *p* < 0.05, && *p* < 0.01, statistically significant difference between CE + DTX-solution and CE + DTX-LNS.

The anti-proliferation effects of CE-LNS (No DTX-LNS), DTX-LNS (No CE-LNS), CE-LNS + DTX-LNS (with DTX-LNS 20 μM or with CE-LNS 10 μM) at different molar ratio and CE + DTX-LNS were evaluated by MTT assay on B16 and MCF-7 cells (Feng et al., 2014). As shown in Figure 4(Ac and f), Blank-LNS showed almost no toxicity (higher than 85%) at the tested concentration which indicated that Blank-LNS was a safe carrier. In comparison with DTX-solution (DTX dissolved in DMSO and diluted with PBS), DTX-LNS demonstrated significant lower half growth inhibition concentration (IC50) values (10.22 ± 1.03 μM versus 43.01 ± 5.41 μM on B16 cells and 16.12 ± 1.47 μM versus 51.94 ± 5.74 μM on MCF-7 cells, *p* < 0.01, respectively), indicating that LNS could significantly enhance the delivery efficiency of payloads. However, as is shown in Figure 4(Aa and e), DTX-LNS alone had a limited effect on killing cancer cells even at high doses. Whereas combining with CE-LNS, the DTX-LNS potently produced cell death at a relatively lower dose, which confirmed that CE-LNS could sensitize DTX-LNS-induced cell death. Similarly, when combining with DTX-LNS, CE-LNS could also induce a significant enhancement of cytotoxicity at some of the experimented concentrations (Figure 4(Aa and d). Notably, when CE-LNS combined with DTX-LNS at a molar ratio of 0.5:1, the strongest cytotoxicity was found on B16 and MCF-7 cells (*p* < 0.01), respectively (Figure 4(Ac and f). According to these results, it was reasonable to deduce that simultaneous delivery of CE and DTX in LNS would maintain the synergistic effect of CE + DTX.

### Apoptosis effect of CE + DTX-LNS

Caspase-3 is one of the crucial mediators of apoptosis, being essential for certain processes associated with the dismantling of the cell and the formation of apoptotic bodies (Choudhary et al., 2015). Therefore, the Caspases-3 activity after different treatments on B16 and MCF-7 cells were studied to quantitatively evaluate whether there existed synergistic apoptotic effect (Figure 4B). Unexpectedly, there was no significant difference on the Caspase-3 level between CE + DTX-LNS and CE + DTX-solution (CE + DTX-S) for 24 h incubation. On the contrary, the Caspase-3 level activated by CE + DTX-LNS was even lower than that of CE + DTX-solution. This strange phenomenon probably caused by the slow release of payloads from CE + DTX-LNS. Therefore, the incubation time was pronged to 48 h. For 48 h incubation, the Caspase-3 activity of cells treated with CE + DTX-LNS was significantly higher than that caused by CE + DTX-solution (CE + DTX-S, *p* < 0.05), DTX-LNS (20 μM, *p* < 0.01) and CE-LNS (10 μM, *p* < 0.01) both on B16 and MCF-7 cells, which further proved the synergistic apoptotic effect of CE + DTX-LNS.

### *In vivo* imaging study

CE + DTX-LNS possesses nanosized structure and lipid protection corona, thus is expected to be beneficial for accumulation in tumor sites through the EPR effect, which is also called passive targeting of nanocarrier delivery system (Liu et al., 2014). To demonstrate this, the *in vivo* biodistribution of CE + DTX-LNS was monitored using a live fluorescence imaging technique. NBD-CE + Rho-DTX-LNS was administrated by tail vein injection to B16 tumor-bearing mice.

**Figure 5.**
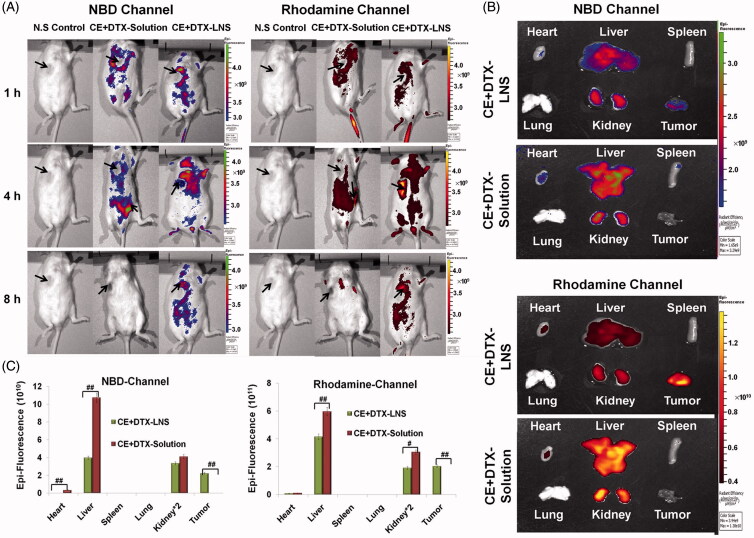
The biodistribution of CE + DTX-LNS by live imaging system *in vivo* and *ex vivo*. (A) Fluorescence images of B16 tumor bearing mice at different time intervals after intravenous administration of CE + DTX-LNS (*n* = 3). Mice administered with NBD-CE and Rho-DTX dual labeled CE + DTX-solution (NBD-CE and Rho-DTX dissolved in DMSO and diluted with PBS) and N.S were served as control. (B) *Ex vivo* fluorescence images of tissues and tumor after intravenous administration of CE + DTX-LNS and CE + DTX-solution. (C) Quantitative analysis for the *ex vivo* tissues and tumor (*n* = 3). # *p* < 0.05, ## *p* < 0.01.

The fluorescence image photographed at predetermined times revealed the biodistribution of both DTX and CE (Figure 5A). For CE + DTX-LNS, the strongest signals were observed at the tumor site, making the tumors easy to be distinguished from normal tissues. The signal intensity at the tumor site increased to the maximum at 4 h and then gradually decreased for the time remaining. More interestingly, when the fluorescence gradually faded from other parts of the body over time, it could still be detected at the tumor site. In contrast, considerable fluorescence signals from the mice treated with CE + DTX-solution were detected in the whole bodies. The strongest fluorescence signals were observed at 1 h after administration, followed by a rapid decrease over time, and the signal completely absent within 8 h. All these results demonstrated that the co-delivered CE + DTX-LNS with appropriate particle size could prolong the circulation time and enhance payloads accumulation in tumor sites.

To accurately evaluate the biodistribution of both DTX and CE, 4 h after intravenously administration, the B16 tumor-bearing mice were sacrificed, and the heart, liver, spleen, lung, kidney and tumor were excised. As shown in Figure 5B, the DTX + CE-solution showed almost no accumulation in tumor site, but relatively high accumulation in liver, kidney and heart, suggesting that the combination delivery of DTX and CE in solution could not promote the tumor uptake. Besides, the low drug content in tumor and high drug content in normal tissues were probably contributed to the high systematic toxicity of free DTX. Differently, the co-delivered CE + DTX-LNS showed high tumor accumulation, which probably due to the EPR effect. According to the fluorescence amounts of the signals (fluorescence intensity threshold was set at 30%), the biodistribution of DTX and CE was further quantified (Figure 5C). These results were consistent with the observation of live imaging, showing that CE + DTX-LNS was mainly accumulated in liver, kidney and tumor. Specifically, in comparison with the mice treated with CE + DTX-solution, the content of DTX and CE were significantly increased in tumor sites (*p *< 0.01) and significantly decreased in liver (*p *< 0.01) or kidney (*p *< 0.05). Therefore, it could be expected that the high tumor accumulation of CE + DTX-LNS was favorable to reduce side effects and enhanced the synergistic antitumor effect *in vivo*.

### *In vivo* anti-tumor study

**Figure 6.**
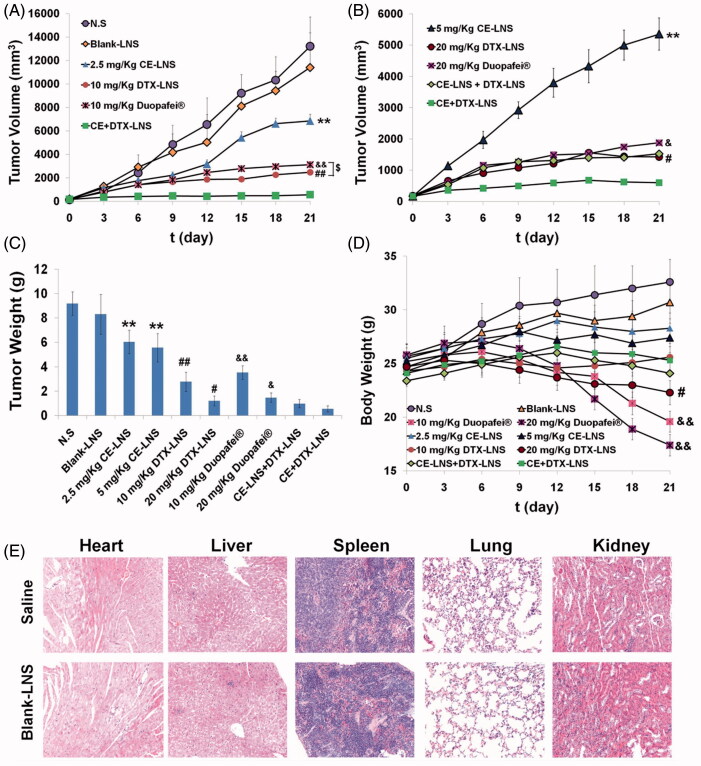
The antitumor effect of CE + DTX-LNS in B16 tumor bearing mice *in vivo* and the histological evaluation. Mice treated with CE-LNS (2.5/5 mg/kg), DTX-LNS (10/20 mg/kg), Duopafei® (10/20 mg/kg), physical mixture of CE-LNS + DTX-LNS, Blank-LNS and N.S were served as controls (*n* = 5). (A) and (B) Tumor volume; (C) Tumor weight; (D) Body weight change. (E) Representative microscopy images of H&E-stained histological sections treated with saline and Blank-LNS (*n* = 3). The magnification was 100. Note: ** *p* < 0.01, statistically significant difference between CE-LNS and CE + DTX-LNS; # *p* < 0.05, ## *p* < 0.01, statistically significant difference between DTX-LNS and CE + DTX-LNS; & *p* < 0.05, && *p* < 0.01, statistically significant difference between Duopafei® and CE + DTX-LNS; $ *p* < 0.05, statistically significant difference between DTX-LNS and Duopafei®.

To evaluate whether the synergistically increased cytotoxicity *in vitro* and enhanced accumulation in tumor sites could lead to synergistic therapeutic efficacy *in vivo*, the anti-tumor effect of CE + DTX-LNS (2.5 mg/kg CE and 10 mg/kg DTX) was performed in B16 tumor-bearing mice. Mice treated with CE-LNS, DTX-LNS, Duopafei®, CE-LNS + DTX-LNS, Blank-LNS and N.S were served as controls. As shown in Figure 6A, by the end of 21 days, the tumor volume of mice treated with 10 mg/kg DTX-LNS were 27.82% of that treated with N.S, which exhibited a considerable tumor inhibition efficacy. Moreover, 10 mg/kg DTX-LNS could achieve a significantly higher antitumor efficacy than that of 10 mg/kg Duopafei® (*p* < 0.05), showing the high delivery efficacy of LNS. CE could further improve the inhibitory effect of DTX, as the highest antitumor activity was achieved when CE and DTX were co-delivered in CE + DTX-LNS. As shown in Figure 6(B and C), with almost completely inhibition of tumor growth (93.94 ± 2.76%), CE + DTX-LNS showed significantly higher tumor inhibition ratio than that of CE-LNS (5 mg/kg, *p* < 0.01), DTX-LNS (20 mg/kg, *p* < 0.05) and Duopafei® (20 mg/kg, *p* < 0.05), respectively, showing promising synergistic antitumor efficacy *in vivo*. Furthermore, at the end of experiment, the tumor inhibition ratio of CE-LNS + DTX-LNS was only 90.44% of these mice treated with CE + DTX-LNS, demonstrating that the physical mixture of CE-LNS and DTX-LNS could not induce a synergistic effect on tumor growth inhibition. The superior therapeutic efficacy of CE + DTX-LNS could be attributed to the high tumor accumulation of payloads, increased cellular uptake of CE and DTX by the same tumor cell and the synergistic effect of CE + DTX on cytotoxicity and apoptosis.

Body weight changes in all mice groups, as an indicator of systemic toxicity, were measured simultaneously (Figure 6D) (Oh et al., 2008). Severe weight loss was seen in mice after the administration with Duopafei®. Among the eight mice injected with Duopafei®, three mice (20 mg/kg) and one mouse (10 mg/kg) died respectively within 21 days, which clearly indicating the high toxic effects of free DTX and/or the solvent system. In contrast, the other 8 mice treated with CE-LNS (2.5/5 mg/kg), DTX-LNS (10/20 mg/kg), CE-LNS + DTX-LNS and CE + DTX-LNS were all alive after 21 days and the body weights increased steadily at similar rates except when tumors grew too big and affected the mice body weights. Therefore, it could be concluded that LNS could increase survival rates by reducing *in vivo* toxicity to normal tissues. During the experiment period, the weight loss induced by CE + DTX-LNS was much lower than those induced by DTX-LNS (20 mg/kg, *p* < 0.05) and Duopafei® (10/20 mg/kg, *p* < 0.01) respectively, displaying significantly lower systematic toxicity.

Overall, the high antitumor efficacy, together with the lack of systemic toxicity, suggested that CE-LNS could act as an ideal nanocarrier for enhanced delivery of DTX to tumor sites and achieve better synergistic antitumor efficacy.

### The histological observation

To evaluate the tissue toxicities and the compatibility of the blank nanocarrier, Blank-LNS were used in the histological observation. A histological analysis of organs (heart, liver, spleen, lung and kidney) was performed to determine whether or not Blank-LNS caused tissue damage, inflammation, or lesions (Luo et al., 2012). As shown in Figure 6E, within the experiment duration, no visible histological changes were observed compared to the N.S control (top row), indicating that Blank-LNS had good biocompatibility *in vivo* and did not produce additional tissue toxicities. This phenomenon was probably due to the composition of LNS, as Blank-LNS was prepared only with injectable soya lecithin and glycerol, which promoted the high security of LNS and the *in vivo* application.

## Conclusion

In this study, a CE-based nanocarrier CE-LNS for enhanced delivery of DTX with synergistic effect was developed by high pressure homogenization. CE + DTX-LNS was spherical particles with small size and negative charge. The stability of CE + DTX-LNS was ensured by lyophilization. DTX was effectively delivered into tumor cells and the sustained released of DTX from CE + DTX-LNS lead to the co-localization of CE and DTX in the same cell. The accumulation of CE and DTX in tumor site by EPR effect was observed *in vivo* and *ex vivo*. Additionally, CE + DTX-LNS exhibited synergistic antitumor effect both *in vitro* and *in vivo*. It is believed that the CE + DTX-LNS possessed the synergistic anticancer efficiency, reduced side effects and the biocompatibility. Furthermore, CE lipid-based nanosuspension holds great potential to be an appropriate cancer therapy agent for CE-based combination therapy.
